# Radiobiology of stereotactic ablative radiotherapy (SABR): perspectives of clinical oncologists

**DOI:** 10.7150/jca.44408

**Published:** 2020-06-27

**Authors:** Shan Li, Liangfang Shen

**Affiliations:** Department of Oncology, Xiangya Hospital, Central South University, No. 87, Xiangya Road, Changsha, Hunan Province 410008, China

**Keywords:** Radiobiology, stereotactic ablative radiotherapy, SABR, oncologist

## Abstract

Stereotactic ablative radiotherapy (SABR) is a novel radiation treatment method that delivers an intense dose of radiation to the treatment targets with high accuracy. The excellent local control and tolerance profile of SABR have made it become an important modality in cancer treatment. The radiobiology of SABR is a key factor in understanding and further optimizing the benefits of SABR. In this review, we have addressed several issues in the radiobiology of SABR from the perspective of clinical oncologists. The appropriateness of the linear-quadratic (LQ) model for SABR is controversial based on preclinical data, but it is a reliable tool from the perspective of clinical application because the biological effective dose (BED) calculated with it can represent the tumor control probability (TCP). Hypoxia is a common phenomenon in SABR in spite of the relatively small tumor size and has a negative effect on the efficacy of SABR. Preliminary studies indicate that a hypoxic radiosensitizer combined with SABR may be a feasible strategy, but so far there is not adequate evidence to support its application in routine practice. The vascular change of endothelial apoptosis and blood perfusion reduction in SABR may enhance the response of tumor cells to radiation. Combination of SABR with anti-angiogenesis therapy has shown promising efficacy and good tolerance in advanced cancers. SABR is more powerful in enhancing antitumor immunity and works better with immune checkpoint inhibitors (ICIs) than conventional fractionation radiotherapy. Combination of SABR with ICIs has become a practical option for cancer patients with metastases.

## Introduction

Stereotactic ablative radiotherapy (SABR) is a novel radiation treatment method that delivers an intense dose of radiation to the treatment targets with a higher dose per fraction (> 5 Gy) and fewer fractions (1-5 fractions) compared with conventional fractionation radiotherapy 1. SABR is also known as stereotactic radiosurgery (SRS) for treating brain lesions and as stereotactic body radiation therapy (SBRT) in the treatment of extracranial tumors 2. Due to its excellent local control and tolerance profile, SABR currently plays a very important role in the treatment of many cancers, such as non-small cell lung cancer (NSCLC), liver cancer, pancreatic cancer, and brain tumors.

The radiobiology of SABR is a key factor in understanding and further optimizing the benefits of SABR 2, 3. However, so far, the majority of publications on radiobiology are based on the perspectives of radiobiologists or researchers of basic science. As clinical oncologists, we are eager to improve patient survival and reduce treatment toxicity with assistance from the radiobiology of SABR. Therefore, in this review, we have addressed several issues in the radiobiology of SABR from the perspective of clinical application, including the appropriateness of linear-quadratic (LQ) model in SABR, the role of hypoxia in SABR, the role of vascular change in SABR, and the synergistic effect of SABR and immune checkpoint inhibitors.

## 1. Is the LQ model appropriate for SABR?

As a biologically based and practical model, the LQ formalism is the most commonly accepted tool for evaluating the relationship between the radiation dose and its biological effects 4. The biological effective dose (BED) deduced from the LQ model has been widely used by clinical oncologists to predict tumor control probability (TCP) and normal-tissue tolerance 5. However, the validity of the LQ model at high dose per fraction (> 5 Gy) is controversial. Some preclinical studies based on either cells or animal models showed that the LQ model failed to accurately predict a high-dose response 2, 6, 7, while other studies showed that the LQ model fitted the *in vitro* and *in vivo* survival data well up to 15-20 Gy per fraction 8, 9. Besides, several modified models with more parameters, such as the universal survival curve (USC) model, the linear-quadratic-linear (LQ-L) model, and the Pade linear quadratic (PLQ) model, have been proposed to replace the LQ model and reported to fit the dose-response curve better at high dose per fraction in preclinical experiments 10-12.

In spite of the controversy in preclinical studies, the most important question for clinical oncologists is whether BED calculated with the LQ model can represent TCP. It is generally accepted that higher BED results in higher TCP until it reaches a plateau. Therefore, a practical method to examine the validity of the LQ model is to investigate the relationship between TCP and BED. Brown et al. analyzed 2696 patients with stage I NSCLC who were treated with 3-dimension conformal radiotherapy (3D-CRT) or SBRT and plotted TCP against the BED calculated with the LQ model. Their results demonstrated that TCP increased monotonically with BED for different SBRT regimens and the data for 3D-CRT also fell on the curve, indicating that the BED calculated with the LQ model can represent TCP for both SBRT and 3D-CRT 13. To further confirm the validity of the LQ model in SABR, several studies compared the LQ model with other models in the fitness of TCP and BED. The LQ-L model and the USC model are the most commonly reported modified models that require additional parameters, as shown in Table 1. Mehta et al. reviewed the data of the same 2696 patients mentioned above and showed that the fitness of TCP and BED was similar for the LQ model and the USC model 14. Guckenberger et al. compared the LQ and LQ-L formula for modeling TCP in 395 stage I NSCLC patients treated with SBRT, and they showed that the fit of the LQ-L model was not significantly better than that of the LQ model 15. Similarly, Santiago et al. analyzed 31 studies that reported 3-year local control in 2319 patients with stage I NSCLC, and they showed that the fit of the LQ and LQ-L models did not differ substantially 16. Moreover, Shuryak et al. analyzed the TCP data of 2965 patients with lung tumors or brain metastases who received SABR, and they showed that the LQ model provided a significantly better fit over the entire range of treatment doses than the LQ-L model and the USC model 17.

It is also worth mentioning that simplicity is an unneglectable property of a model in clinical application. As shown in Table 1, α and β are the only parameters required for the LQ model, while other modified models require extra parameters. To obtain more reliable data, clinicians generally calculate α and β with statistical methods based on TCP from clinical studies, which is very complicated and has great heterogeneity 18. All of the modified models require additional parameters, which makes it much more difficult in clinical practice. Therefore, the LQ model is simplest of all of the models.

To summarize, the LQ model is an appropriate model for SABR as it can represent TCP and it performs better than or equivalent to the other modified models. As per George Box's well-worn aphorism: 'All models are wrong, but some are useful,' the LQ model is a very useful tool for SABR in spite of the controversy.

## 2. The role of hypoxia in SABR

Due to the oxygen enhancement effect, it is well known that tumor hypoxia correlates with treatment failure after radiotherapy of conventional fractionation regimens. With respect to SABR, because reoxygenation decreases as a result of fewer fractions, the influence of hypoxia is supposed to be more powerful theoretically. However, the high local control rate by SABR seems to attenuate the importance of oxygen. Several issues regarding hypoxia in SABR are discussed below from the perspective of clinical oncologists.

### 2.1 Is hypoxia a common phenomenon in SABR?

Generally, SABR is only suitable for tumors with a small size due to the dose constraints of nearby normal tissues. For example, SABR is commonly recommended for tumors smaller than 5 cm in size for early-stage NSCLC according to the NCCN guidelines (version 1.2020). Hypoxia may not be a common phenomenon in SABR because experiments on animal models have shown that smaller tumors may be less likely to become hypoxic 19, 20. However, other studies have indicated that tumor hypoxia is independent of tumor size 21. Hockel et al. measured the hypoxic status of 103 cervical cancer patients with a computerized polarographic electrode system, and they found that tumor oxygenation was independent of tumor size 22. Similarly, Le et al. measured pO_2_ in 20 patients with resectable NSCLC (median tumor volume 10.8 ml) intraoperatively using the Eppendorf polarographic electrode and showed that tumor hypoxia existed in 19 patients and the severity of hypoxia was independent of tumor volume 23. Moreover, with modern hypoxia imaging techniques, such as the positron emission tomography (PET), we can examine the tumor hypoxia status directly during SABR. Kelada et al. performed dynamic ^18^F-fluoromisonidazole PET-CT during the SBRT treatment in 6 NSCLC patients, and among them, 4 patients showed tumor hypoxia before and during the course of SBRT 24. In addition, another study by Qian et al. also showed imageable hypoxia in 6 of 16 early-stage NSCLC patients treated with SABR by performing ^18^F- pentafluorinated etanidazole PET 25. Therefore, hypoxia is a common phenomenon in SABR in spite of its relatively small tumor size.

### 2.2 Is hypoxia a neglectable factor in determining the efficacy of SABR?

As hypoxia is a common phenomenon in SABR, it is necessary to evaluate its influence on the efficacy of SABR. For radiotherapy with conventional fractionation, it is well accepted that hypoxia is a major factor for tumor radioresistance 26. With respect to SABR, a study by Carlson et al. showed that hypoxia caused a more significant decrease in cell killing during SABR compared to the conventional fractionation regimen in cell lines of prostate cancer and head and neck cancer 27. Another study in animal models also showed that tumors with lower pO_2_ had decreased TCP after SRS 28. With respect to clinical evidence, Goodman et al. reviewed 682 brain metastases treated with SRS and found that 1-year freedom from progression (FFP) probabilities for homogeneously-, heterogeneously-, and ring-enhancing lesions were 90%, 76%, and 57%, respectively, which indicated that hypoxic tumor cells in the necrotic regions were associated with radioresistance 29. Further, Qian et al. also found that imageable hypoxia was associated with worse overall survival (OS), regional failure, and distant failure in 16 early-stage NSCLC patients who were treated with SBRT 25. In addition, Jensen et al. analyzed 162 meningioma patients who received SRS, and they found that the expression of hypoxia-inducible factor 1α (HIF-1α), which is an endogenous marker of hypoxia, was correlated with worse local control after SRS (p=0.046) 30. Taken together, hypoxia has a negative effect on the efficacy of SABR.

### 2.3 Is hypoxic modification necessary in SABR?

Based on the information presented above, it is reasonable to investigate the role of hypoxic modification in SABR. As is already known, the most extensively investigated approach for hypoxic modification is hypoxic radiosensitizer. For conventional fractionation radiotherapy, hypoxic radiosensitizer combined with radiotherapy has provided promising benefits in TCP and OS in cervical cancer and head and neck cancer 26. With respect to SABR, preliminary studies have indicated that SABR plus hypoxic modifiers may be a feasible strategy. A study by Wittenborn et al. showed that hypoxic modifiers (nimorazole, nicotinamide, carbogen breathing, and OXi4503) effectively improved the treatment outcome in a preclinical tumor model treated with stereotactic radiation schedules 31. The RTOG study 95-02, which is a phase Ib clinical trial, showed that SRS combined with etanidazole (a hypoxic cell radiosensitizer) at a dose of 12 g/m^2^ was well tolerated by patients with brain tumors and brain metastases 32. In addition, a phase I clinical trial (NCT03824327) is currently evaluating the safety and efficacy of papaverine hydrochloride (a radiosensitizer targeting mitochondrial respiration) combined with SBRT in treating early-stage NSCLC patients. Other hypoxic modification approaches, such as the increase in oxygen availability through hyperbaric oxygen, dose escalation for hypoxic tumor volume, and adoption of higher linear energy transfer radiation have less impact on general clinical practice and their combination with SABR has seldom been reported. To summarize, preliminary studies indicate that a hypoxic radiosensitizer combined with SABR may be a feasible strategy, but so far there is not adequate evidence to support its application in routine practice.

From the perspective of clinical application, a key question in further improving the efficacy of this combination strategy is identifying the appropriate patients. It has been indicated that the hypoxia status is an important factor in determining the efficacy of hypoxic radiosensitizers when combined with radiotherapy. Toustrup et al. classified tumors as “more” and “less” hypoxic according to the expressions of hypoxia responsive genes, and they found that only patients with “more” hypoxic tumors obtained survival benefits from hypoxic modification of radiotherapy 33. Similar conclusions were reported in the subgroup analysis of the results of the IAEA-HypoX trial, which was an international multicenter randomized trial aimed at investigating the efficacy of combining nimorazole with radiotherapy in head and neck cancer 34. In addition, Yang et al. also found that tumor hypoxia status could predict benefits from hypoxic modification for bladder cancer patients receiving radiotherapy 35. Currently, there are several noninvasive hypoxia imaging techniques available in clinical practice, such as the ^18^F-fluoromisonidazole PET, the ^18^F-fluoroazomycin arabinoside PET, the oxygen-enhanced magnetic resonance imaging (MRI), and the blood oxygen level dependent (BOLD) MRI 36, 37. Further, it should be noted that the status of hypoxia in tumors can change during the treatment course of SABR 24. Therefore, monitoring the status of tumor hypoxia during the treatment course of SABR and selecting patients with hypoxic tumors as the candidates is a potential strategy for further improving the efficacy of combining SABR with hypoxia modifiers.

## 3. The role of vascular change in SABR

### 3.1 The vascular change in SABR

It is well known that the intratumor microenvironment has a great influence on the oncogenesis, invasion, and metastasis of tumor cells. As an important part of the microenvironment, tumor microvasculature plays a key role in providing tumor cells with oxygen and nutrients. Therefore, it is necessary to investigate the vascular change in SABR in order to understand the radiobiology better. Park et al. analyzed 43 representative studies on radiation-induced tumor vascular change and found that although the reported results were inconsistent, they could be generalized as follows. For conventional fractionation radiotherapy (< 3 Gy per fraction), the morphology and function of vasculature and the blood perfusion were not impaired until the end of the treatment, which was attributed to the declined demands for nutrients and oxygen as a result of radiation-induced tumor cell death. For SABR regimens (> 5 Gy per fraction), irradiation of 5-10 Gy in a single dose caused relatively mild decrease in tumor blood flow, but irradiation of higher than 10 Gy per fraction induced severe and rapid blood perfusion reduction, which was attributed to the damage of the integrity and viability of vascular endothelial cells by irradiation 38. Similar results have been reported by several other studies later, which adopted different methods to measure the change of vasculature in animal models 39-41.

A key question regarding the SABR induced endothelial apoptosis and blood perfusion reduction is whether it affects the response of the tumor to radiotherapy. As is well known, irradiation can lead to direct cancer cell death through DNA damage. Can the vascular change cause indirect cancer cell death in SABR by depriving the supply of oxygen and nutrients? Kocher et al. developed a 3-dimensional computer simulation method to determine the factors affecting the tumor response to radiotherapy, and they showed that the therapeutic effect of SRS in brain tumors cannot be explained without the consideration of vascular effects 42. Similarly, Monica et al. proposed that the tumor response to radiotherapy was regulated by endothelial cell apoptosis. In their study, the MCA/129 fibrosarcomas transplanted on endothelial apoptosis resistant mice displayed markedly reduced baseline microvascular endothelial apoptosis and were resistant to single-dose radiation up to 20 Gy 43. Later, Moeller et al. suggested that endothelial apoptosis contributed more significantly to tumor cell death in single dose radiation (> 8-10 Gy) than conventional fractionation regimens (1.8-3 Gy/fraction), because the death signaling pathway in endothelium was repressed by the activation of HIF-1α during the process of hypoxia/reoxygenation 44, 45. Therefore, endothelial apoptosis and blood perfusion reduction in SABR may enhance the response of tumor cells to radiation.

### 3.2 The combination of anti-angiogenic therapy and SABR

Anti-angiogenic therapy, which focuses on inhibiting neovascularization or endothelial cell function, has currently become an important strategy in cancer treatment 46, 47. It has been reported that the normalization of vascular flow by anti-angiogenesis drugs can reverse the hypoxia and low pH in the tumor microenvironment, thus improving the radiosensitivity of cancer cells 48. Combination of anti-angiogenic therapy with conventional fractionation radiotherapy has been reported to be a promising strategy in several clinical trials 49, 50. With respect to SABR, many studies are investigating the safety and efficacy of its combination with different types of anti-angiogenesis drugs, as shown in Table 2.

Bevacizumab is one of the most widely used anti-angiogenesis drugs in cancer treatments. Although bevacizumab plus conventional regimen raidotherapy for the newly diagnosed glioblastoma obtained negative results in two well-known randomized controlled trials 51, 52, bevacizumab plus SRS for recurrent or progressive glioblastoma showed a promising clinical outcome with good tolerance. Morris et al. retrospectively reviewed 45 recurrent glioblastoma patients treated with SRS plus bevacizumab and showed a satisfying median progression free survival (PFS) of 5.3 months and a median OS of 13.3 months without radiation-related adverse events 53. A phase II clinical trial (NCT02120287) with 16 recurrent or progressive glioblastoma patients who underwent SRS plus bevacizumab also achieved a promising outcome with a median OS of 11.7 months and a 6-month PFS of 56.2%. Similar results have been reported in several other restrospective studies or prospective clinical trials with a small sample size 54-56. Prospective studies with a larger sample size and a longer follow-up time are needed to further confirm the efficacy and safety of this combination stategy. With respect to extra-cranial lesions, Mazzola et al. retrospectively reviewed 40 lung metastases of 23 colon cancer patients who underwent SBRT with or without bevacizumab, and they showed that 1-year local control rate in Bevacizumab-group was 93% versus 86% in No-Bevacizumab group and no toxicity superior or equal to grade 3 was recorded in both groups at the time of the analysis 57. Similarly, a phase II clinical trial (NCT01569984) is investigating the combination of bevacizumab with SBRT for treating colorectal liver metastases.

Sorafenib, a multi-target inhibitor that targets the vascular endothelial growth factor receptor (VEGFR)/platelet-derived growth factor (PDGFR) pathway in tumor vasculature and the RAF/MEK/ERK pathway in tumor cells, has been widely used in the treatment of advanced liver cancer and renal cancer 58-60. Preclinical experiments have shown that SBRT plus sorafenib improved the outcome for advanced hepatocellular carcinoma with acceptable toxicity 61, 62. A phase I clinical trial by Brade et al. showed that the combination of SBRT with sorafenib in locally advanced liver cancer patients achieved a promising response rate (36%-50%), but with significant toxicity in patients with highly irradiated liver volume (30%-60%) 63. In addition, a phase III clinical trial is currently comparing sorafenib plus SBRT and sorafenib alone for treating primary liver cancer (NCT01730937). Sunitinib, another multi-target inhibitor that inhibits several targets, including VEGFR1, VEGFR2, and VEGFR3, has been approved for the treatment of renal cancer, pancreatic cancer, and gastrointestinal stromal cancer. The combination of sunitinib with SABR has also been reported as a feasible strategy in several phase I/II clinical trials. A phase II study (NCT00463060) showed that SBRT plus sunitinib achieved a durable clinical response with manageable toxicity in a subset of cancer patients with oligometastases 64. Another phase I/II trial showed that kidney and prostate cancer patients with oligometastases who received SBRT plus sunitinib achieved a significantly improved OS (hazard ratio = 0.25, p = 0.04) with manageable toxicity in most of the cases 65. Further, a phase II trial showed that SRS plus adjuvant sunitinib exhibited an acceptable safety profile and a comparable PFS to SRS plus WBRT in patients with 1-3 brain metastases 66.

In addition, several other studies are also investigating the combination of SABR with other anti-angiogenesis drugs, such as pazopanib 67, apatinib (NCT03356600), vandetanib (NCT00822887), and Endostar 68. To summarize, combining SABR with anti-angiogenic therapy has showed promising efficacy and good tolerance in advanced cancers, and more prospective studies are needed to further confirm its efficacy and toxicity.

### 3.3 Issues to be solved in clinical application

From the perspective of clinical application, there are several practical issues remained to be solved regarding the combination of SABR with anti-angiogenic therapy, including the optimal timing of combination, the optimal dose/fractionation regimen of SABR, the optimal anti-angiogenesis agent, and the appropriate patients. Unfortunately, so far, the available data is too limited to draw definite conclusions regarding these issues. However, based on exsiting preclinical and clinical studies, we can make a hypothesis regarding the timing of combination. Preclinical studies have demonstrated that anti-angiogenesis agents cause a transient increase in blood perfusion by normalizing the abnormal tumor vasculature in the acute phase (1st to 3rd day after administration), followed by a significant reduction in blood perfusion resulted from the decreased tumor microvessel density on the 5th to 7th day 69, 70. As a result, hypoxia in the tumor microenvironment, which is detrimental for the radiosensitivity, is alleviated in the acute phase. Therefore, the acute phase of anti-angiogenesis agents would be the optimal timing for SABR. Several clinical studies, in which SABR was performed shortly after the start of the administration of anti-angiogenesis agents, have obtained satisfying clinical outcomes 54, 63, 65. In addition, for recurrent malignant glioma patients who received SRS and bevacizumab, the median OS was 14.4 months in a study in which SRS was performed immediately after the administration of bevacizumab 54, while the median OS was 10 months in another study in which SRS was performed before the administration of bevacizumab 56. The results of these studies indicate that performing SABR shortly after the start of the administration of anti-angiogenesis agents is the opitmal timing, but more convincing evidence from well-designed prospective stuides are needed to further confirm it.

## 4. Synergistic effect of SABR and immune checkpoint inhibitors

Recently, the emergence of immune checkpoint inhibitors (ICIs) has changed the patterns of cancer treatment. A variety of preclinical and clinical studies have indicated that combining ICIs with SABR can induce a synergistic effect. A typical case of this synergistic effect was reported by Michael et al., in which SABR (28.5 Gy/3 F) reversed the acquired resistance to ipilimumab and achieved disease responses both inside and outside of the radiation field in a patient with recurrent and unresectable melanoma 71. Several issues regarding the synergistic effect are discussed below from the perspective of clinical oncologists.

### 4.1 What is the mechanism for the synergistic effect?

It has been reported that radiotherapy can enhance tumor specific immunity via multiple mechanisms, including increasing the release of tumor associated antigens from irradiation induced dying cancer cells, enhancing the recruitment and activation of antigen presenting cells, promoting the priming of tumor specific T-cells, and inducing the release of cytokine and chemokines, such as type I/II interferons and complements 72, 73. However, radiotherapy also has a negative impact on the immune system as leukocytes are highly sensitive to radiation and lymphopenia is a common phenomenon during the treatment of radiotherapy. In addition, the immune suppressive cells and inhibitory cytokines within the tumor microenvironment, such as the myeloid-derived suppressor cells, T regulatory (Treg) cells, transforming growth factor-β, and interleukin-10, can also induce local immune suppression 74. Therefore, combination of radiotherapy with immunotherapy is a promising strategy for augmenting the antitumor immune response. As is well known, the most common type of immunotherapy in clinical practice is ICIs, such as the cytotoxic T lymphocyte-associated antigen 4 (CTLA-4) inhibitors (e.g., ipilimumab) and the programmed cell death 1/ programmed cell death ligand 1 (PD1/PD-L1) inhibitors (e.g., pembrolizumab, nivolumab, and durvalumab). A study by Dovedi et al. showed that fractionated radiotherapy caused PD-L1 upregulation on tumor cells in mouse models and combination of PD1/PD-L1 inhibitors with radiotherapy generated efficacious CD8 T-cell responses and better tumor control rate 75. Similarly, another study by Sharabi et al. also showed that combining PD1 inhibitors with radiotherapy resulted in increased tumor antigen-specific T cell- and B cell-mediated immune responses in animal models 76. In addition, a preclinical experiment showed that anti-CTLA4 inhibited Treg cells and increased the CD8 T-cell to Treg (CD8/Treg) ratio, while radiotherapy enhanced the diversity of the T-cell receptor (TCR) repertoire. Combination of anti-CTLA4 with radiotherapy promoted the expansion of cytotoxic T cells with an extended TCR repertoire 77.

It is noteworthy that the synergistic effect of radiotherapy with ICIs seems to be more commonly reported in SABR than conventional fractionation radiotherapy. Is SABR more powerful in enhancing antitumor immunity? On one hand, some studies have shown that a higher dose per fraction creates greater tumor immunogenicity. Schaue et al. irradiated mice bearing B16-OVA murine melanoma with a single dose of 5 Gy, 7.5 Gy, 10 Gy, or 15 Gy, and they showed that tumor-reactive T cells increased with the size of the radiation dose 78. Moreover, an animal model experiment by Lan et al. compared the synergistic effect of anti-PDL1 with two fractionation regimens of the same BED (23 Gy/2 F vs 36 Gy/9 F) and showed that the 23 Gy/2 F group achieved better tumor control and OS than the 36 Gy/9 F group 79. On the other hand, focused irradiation field of SABR resulted in less damage to the immune system. A study by Ladbury et al. evaluated the estimated dose of radiation to immune cells (EDRIC) in 117 patients with stage III NSCLC who received definitive radiotherapy. Their results showed that a higher EDRIC was correlated with greater risk of grade ≥3 lymphopenia (P = 0.004) and EDRIC was independently associated with OS (HR 1.17, P = 0.03), local PFS (HR 1.17, P = 0.02), and disease free survival (DFS) (HR 1.15, P = 0.04) 80. Therefore, SABR is more powerful in enhancing antitumor immunity and works better with ICIs than conventional fractionation radiotherapy.

### 4.2 How is the efficacy and safety of the ICI-SABR combination in clinical practice?

As is already known, the prognosis of most advanced cancers is not satisfactory 81-83. ICIs have obtained a promising clinical outcome in the treatment of many cancers, especially in NSCLC and melanoma 84-86. SABR is commonly used as a palliative modality for advanced cancer with intent to achieve local control of metastatic lesions, such as brain and bone metastases. Based on the mechanism mentioned above, the ICI-SABR combination is expected to improve both local and systemic responses.

NSCLC is the most common type of cancer as well as a main cause of cancer-associated death worldwide 83, 87. With the emergence of ICIs, the treatment of metastatic NSCLC has changed dramatically, as several well-known randomized controlled trials, such as CheckMate017 88, CheckMate057 89, and KEYNOTE010 90, have obtained inspiring results. Meanwhile, SABR has also been regarded as an effective local treatment option for NSCLC with oligometastases 91. Therefore, combining ICI with SABR is a strategy worth trying for treating advanced NSCLC. A phase 2 randomized trial (PEMBRO-RT) of 76 patients with recurrent or metastatic NSCLC showed that SBRT plus pembrolizumab achieved a higher overall response than pembrolizumab alone (36% vs 18%) without an increase in treatment-related toxicity 92. In addition, Rodolfo et al. recently published a systemic review, which analyzed 1736 metastatic NSCLC patients in 6 phase I-II prospective studies and 12 retrospective studies from 2009 to 2019. Their results showed that SABR combined with anti-PD1, anti-PDL1 or anti-CTLA4 drugs obtained high rates of local control (71%) and distant/abscopal response (41%) with a good safety profile 93. To further confirm the efficacy and safety of this strategy, several phase II and phase III clinical trials are ongoing, including NCT02492568, NCT03955198, and NCT03867175. In addition, SABR plus dual ICIs (anti PD1/PDL1 and anti-CTLA4) is also under investigation in a phase I clinical trial for advanced NSCLC (NCT03275597). It is worth mentioning that the ICI-SABR combination is also a promising strategy for early-stage NSCLC. It is well known that for early-stage NSCLC, SABR is a standard treatment option for patients who are medically inoperable or who refuse surgery. However, a main failure pattern of early-stage NSCLC is distant metastasis, and systemic treatment with good tolerability may be needed 94. Therefore, the combination of SABR with ICI in early-stage NSCLC is a potential option, and several clinical trials are currently ongoing to investigate the feasibility of this strategy, as shown in Table 3.

Melanoma is another type of cancer which showed inspiring benefits from treatment with ICIs. Nowadays, ICIs have become the first-line treatment option for unresectable or metastatic melanoma. However, prognosis of advanced melanoma remains very poor, even after treatment with ICIs 86. Therefore, the combination of ICI with SABR is under investigation to determine its feasibility. Murphy et al. analyzed 26 patients with metastatic melanoma who received ICIs (pembrolizumab, nivolumab, and/or ipilimumab) plus SRS for brain metastases and showed a favorable median survival of 26.1 months compared with historical controls without grade 4-5 toxicity 95. Similarly, Minniti et al. retrospectively reviewed 80 melanoma patients with brain metastases who received SRS plus ipilimumab/nivolumab and showed meaningful intracranial control (6-month PFS 48-69%, 12-month PFS 17-42%) 96. In addition, Diao et al. reviewed 91 melanoma patients treated with SRS for brain metastases and showed that patients who received ipilimumab had better OS than patients who did not received ipilimumab (median OS 15.1 months vs 7.8 months, p = 0.02) 97. Besides, similar results have been reported in some other retrospective studies 98-100. Therefore, ICI-SABR is a promising strategy for metastatic melanoma and several phase I/II clinical trials are underway to further confirm the safety and efficacy of this combination (NCT03354962, NCT02858869, and NCT02716948).

In addition, clinical trials of SABR plus ICIs are also under investigation for several other cancers, including head and neck cancer, breast cancer, liver cancer, pancreatic cancer, genital cancer, Merkel cell cancer, and soft tissue cancer, as listed in Table 3. To summarize, SABR plus ICIs has become a practical option for patients with metastases, especially for those with NSCLC and melanoma.

### 4.3 Issues to be solved in clinical application

From the perspective of clinical application, there are several practical issues remained to be solved regarding the ICI-SABR combination, especially the optimal sequence of combination and the optimal dose/fractionation regimen of SABR.

With respect to the sequence of combination, there are three modes under investigation: SABR followed by ICI, ICI followed by SABR, and concurrent SABR and ICI. As mentioned above, SABR can enhance anti-tumor immunity via multiple mechanisms, while ICI can enhance the efficacy of SABR by overcoming radiation-induced immunosuppression. Theoretically, concurrent SABR and ICI would be the best mode to obtain the synergistic effect, and this hypothesis is supported by several studies. Pinnamaneni et al. reviewed the survival outcomes of metastatic lung cancer patients who received nivolumab and SABR, and they found that patients receiving SABR during nivolumab treatment had significantly better OS than patients receiving SABR followed by nivolumab 101. In addition, a randomized controlled study compared the efficacy of SABR followed by ipilimumab and SABR alone in 799 patients with metastatic prostate cancer, and no survival differences were observed between the two groups, indicating that SABR followed by ipilimumab was not an effective combination strategy 102. Further, Chen et al. analyzed 260 cancer patients who had brain metastases treated with SRS, and they found that concurrent SRS and ICI was associated with a better OS compared with non-concurrent SRS and ICI (HR 2.40, p = 0.006) 99.Similar results have been reported in several other retrospective studies 103, 104. It is worth mentioning that a major concern about the strategy of concurrent SABR and ICI is the safety issue, as radiotherapy and ICI may result in overlapping toxicities, such as the pneumonitis. However, evidence from retrospective studies and preliminary results of prospective studies has indicated that concurrent radiotherapy and ICI is tolerable. A phase 2 clinical study showed that concurrent radiotherapy with nivolumab was safe and tolerable regarding the 6-month rate of pneumonitis grade ≥ 3 for NSCLC 105. A meta-analysis of 17 clinical studies showed that that concurrent SRS and ICI did not increase the overall incidence of radionecrosis than the non-concurrent group 104. Taken together, concurrent SABR and ICI is the optimal sequence of combination, but more prospective stuides are needed to further confirm its efficacy and safety.

With respect to the optimal dose/fractionation regimen for the ICI-SABR combination, there is not enough data available to draw a definite conclusion, but the BED seems to be a potential reference for selecting appropriate regimens. A meta-analysis of the abscopal effect in preclinical models indicated that a SABR regimen with higher BED was more likely to trigger the abscopal effect, and a BED of 60 Gy resulted in a probability of 50% in generating abscopal effects 106. In addition, Foster et al. analyzed 44,498 patients with stage IV NSCLC from the National Cancer Database, and their results showed that for patients receiving SABR and immunotherapy, a SABR regimen with BED higher than 60Gy was associated with better OS 107. Further, Bang et al. retrospectively reviewed 133 patients with metastatic NSCLC, melanoma, or renal cell cancer who received ICI treatment and hypofractionation radiotherapy, and they found a significant association between increased BED and immune-related adverse events (p = 0.01) 108. Therefore, taking BED as a reference to identify the appropriate dose/fractionation regimens with good efficacy and tolerability may be a feasible strategy.

## 5. Conclusions

To summarize, the appropriateness of the LQ model for SABR is controversial based on preclinical data, but it is a reliable tool from the perspective of clinical application because the BED calculated with it can represent the TCP. Hypoxia is a common phenomenon in SABR in spite of its relatively small tumor size and has a negative effect on the efficacy of SABR. Preliminary studies indicate that a hypoxic radiosensitizer combined with SABR may be a feasible strategy, but so far there is not adequate evidence to support its application in routine practice. The vascular change of endothelial apoptosis and blood perfusion reduction in SABR may enhance the response of tumor cells to radiation. Combination of SABR with anti-angiogenesis therapy has showed promising efficacy and good tolerance in advanced cancers. SABR is more powerful in enhancing antitumor immunity and works better with ICIs than conventional fractionation radiotherapy. Combination of SABR with ICIs has become a practical option for cancer patients with metastases.

## Figures and Tables

**Table 1:** LQ model, USC model and LQ-L model

Model	Parameters	BED calculation
LQ model 109	α, β	BED_ LQ_ = n*d* (1 + 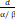 )
USC model 14, 109	α, β, Dq, D_0_, D_t_	BED_ USC_ = n 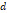 (1 + 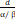 ), 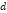 <D_t_ BED_ USC_ = 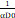 (n 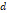 - nDq), 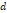 ≥ D_t_
LQ-L model 15, 16	α, β, D_t_	BED_ LQL_ = n 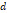 (1 + 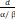 ), 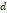 <D_t_ BED_ LQL_ = nD_t_ (1 + 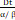 ) +n ( 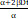 )( 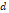 - D_t_), 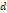 ≥ D_t_

n = number of treatment fractions, *d* = dose per fraction.

**Table 2:** Registered clinical trials of combining SABR with anti-angiogenesis therapy

Trial ID	Anti-angiogenesis drugs	Study design	Patients	Interventions	Primary endpoint
NCT02313272	Bevacizumab	Phase I	Recurrent high-grade gliomas	SABR + Bevacizumab + Pembrolizumab	Safety and tolerability
NCT02829931	Bevacizumab	Phase I	Recurrent high-grade gliomas	SABR + Bevacizumab + Nivolumab + Ipilimumab	Safety and tolerability
NCT01392209	Bevacizumab	Phase I	Recurrent high-grade gliomas	SABR + Bevacizumab	Maximum tolerated dose of SABR
NCT02672995	Bevacizumab	Phase I	Brain metastases	SABR + Bevacizumab	Maximum tolerated dose of SABR
NCT01569984	Bevacizumab	Phase II	Colorectal liver metastasis	SBRT+ Bevacizumab	Tumor perfusion
NCT02120287	Bevacizumab	Phase II	Recurrent or progressive glioblastoma	SRS+ Bevacizumab	OS
NCT01005875	Sorafenib	Not Applicable	Unresectable hepatocellular carcinoma	SBRT + Sorafenib	Safety and tolerability
NCT01276210	Sorafenib	Phase I	Brain Metastases	SBRT + Sorafenib	Maximum tolerated dose of Sorafenib
NCT00672178	Sorafenib	Phase I/II	Metastatic, recurrent, or unresectable renal cell cancer	SBRT + Sorafenib	Response rate
NCT01730937	Sorafenib	Phase III	Primary liver cancer	Arm A: Sorafenib Arm B: SBRT + Sorafenib	OS
NCT00910039	Sunitinib	Phase II	Newly diagnosed brain metastases	SRS + Sunitinib	PFS
NCT00981890	Sunitinib	Phase I	Brain Metastases	SRS + Sunitinib	Maximum tolerated dose of Sunitinib
NCT00463060	Sunitinib	Phase I/II	Limited extent metastatic cancer	SABR+ Sunitinib	Dose limiting toxicity
NCT02019576	Sunitinib	Phase II	Metastatic kidney cancer	SABR + Sunitinib	Local control of metastases
ChiCTR-ONC-13003720	Endostar	Phase II	Advanced pancreatic cancer	SABR + Endostar + Capecitabine	PFS
NCT03356600	Apatinib	Phase II	Brain metastases	SABR + Apatinib	PFS of intracranial lesions
NCT00822887	Vandetanib	Phase I	Recurrent malignant gliomas	SABR + Vandetanib	Safety and tolerability

**Table 3:** Ongoing clinical studies of combining SABR with immune checkpoint inhibitors on ClinicalTrial.gov

	Registration ID	Study design	Patients	Interventions	Primary endpoint
NSCLC	NCT02599454	Phase I	Stage I NSCLC	SBRT+ Atezolizumab	Maximum tolerated dose
	NCT03574220	Phase I	Medically Inoperable Early Stage NSCLC	SBRT + Pembrolizumab	Percent of patients tolerant to Pembrolizumab
	NCT02599454	Phase I	Stage I NSCLC	SBRT + Atezolizumab	Maximum tolerated dose
	NCT03050554	Phase I/II	Early Stage NSCLC	SBRT + Avelumab	Incidence of adverse events
	NCT03383302	Phase I/II	Stage I/II NSCLC	SBRT+ Nivolumab	Lung toxicity (pneumonitis)
	NCT03446911	phase I/II	Stage I NSCLC (planned for surgery)	Arm A: SABR (Prior to surgery) Arm B: SABR + Pembrolizumab (Prior to surgery)	Incidence and severity of adverse events
	NCT02904954	Phase II	Stage I (tumors > 2cm)/ II/ IIIA NSCLC	Arm A: Durvalumab + surgery Arm B: SBRT + Durvalumab + surgery	DFS
	NCT03110978	Phase II	Stage I-IIA or Recurrent NSCLC	Arm A: SBRT + Nivolumab Arm B: SBRT	Event-free survival
	NCT03924869	phase III	Stage I or IIA NSCLC	Arm A: SBRT (45-54Gy/3-5F) +Pembolizumab Arm B: SBRT (45-54Gy/3-5F) +Placebo	Event-free survival
	NCT03833154	phase III	Unresected stage I/II, lymph node negative (T1-3N0M0) NSCLC.	Arm A: SBRT + Durvalumab Arm B: SBRT+ Placebo	PFS
	NCT03275597	Phase I	Oligometastatic NSCLC	SBRT + Durvalumab+Tremelimumab	Incidence of adverse events
	NCT02492568	Phase II	Advanced NSCLC	Arm A: Pembrolizumab Arm B: SBRT + Pembrolizumab	Overall response rate (ORR)
	NCT03955198	phase II	NSCLC patients with 1 to 4 brain metastases	Arm A: SABR Arm B: SABR + Durvalumab	Time to intra-cranial progression
	NCT03867175	phase III	Stage IV NSCLC	Arm A: SBRT +Pembolizumab Arm B: Pembolizumab	PFS
Melanoma	NCT03354962	phase I/II	Melanoma with extra-cranial metastases	Arm A: Nivolumab + Ipilimumab alone Arm B: SBRT with Nivolumab + Ipilimumab	Phase I: Dose Limiting Toxicities incidence. Phase II: PFS
	NCT02858869	Phase I	Melanoma or NSCLC with brain metastases	Arm A: Pembrolizumab+SRS (6Gy) Arm B: Pembrolizumab+SRS (9Gy) Arm C: Pembrolizumab+SRS (18-21Gy)	Incidence of adverse events
	NCT02716948	Phase I	Melanoma with brain or spine metastases	SABR + Nivolumab	Incidence of serious adverse events
Head and neck cancer	NCT03522584	phase I/II	Recurrent/Metastatic squamous cell carcinomas of the head and neck	SBRT + Tremelimumab + Durvalumab,	Incidence of adverse effects
	NCT03546582	Phase II	Recurrent or second primary head and neck squamous cell carcinoma	Arm A: SBRT Arm B: SBRT + Pembrolizumab	PFS
	NCT03749460	phase I/II	Salivary gland cancers	SBRT + Nivolumab + Ipilimumab	Incidence of adverse events
	NCT03539198	Not applicable	Recurrent/Progressive locoregional or metastatic head and neck Cancer	Nivolumab+ proton SBRT	ORR
	NCT03618134	Phase I/II	HPV positive oropharyngeal squamous cell caner	Arm A: SBRT + Durvalumab + TORS + neck dissection Arm B: SBRT + Durvalumab + Tremelimumab + TORS + neck dissection	Phase I: Incidence of adverse events Phase II: PFS
	NCT03402737	Not Applicable	Recurrent squamous cell carcinoma of head and neck	Nivolumab + SBRT (2*6-8Gy, 3*6-8Gy, 3*6-10Gy ,3*6-12Gy)	Maximum tolerated dose of SBRT
Breast cancer	NCT03464942	Phase II	Advanced triple negative breast cancer	Arm A: SABR (20Gy * 1F) + Atezolizumab Arm B: SABR (8Gy * 3F) + Atezolizumab	PFS
	NCT03449238	Phase I/II	Metastatic breast cancer with at least 2 brain metastases	SRS + Pembrolizumab	Tumor response for non-irradiated brain lesions
Liver cancer	NCT03817736	Phase II	Hepatocellular carcinoma	SBRT + TACE + ICI	Number of patients amendable to curative surgical interventions
	NCT03203304	Phase I	Unresectable hepatocellular carcinoma	Arm A: SBRT + Nivolumab Arm B: SBRT+ Nivolumab+ Ipilimumab	Incidence of adverse events
Pancreatic cancer	NCT03599362	Phase II	Locally advanced unresectable pancreatic cancer	SBRT + Nivolumab + Cabiralizumab	Incidence of adverse events
	NCT03716596	Phase I	Late stage or recurrent pancreatic cancer patients	SBRT +Pembolizumab	OS
	NCT02311361	Phase I/II	Unresectable pancreatic cancer	Arm A1: Durvalumab + SBRT (8Gy * 1F) Arm A2: Durvalumab + SBRT (5Gy * 5F) Arm B1: Tremelimumab+ SBRT (8Gy * 1F) Arm B2: Tremelimumab+ SBRT (5Gy * 5F) Arm C1: Tremelimumab+ Durvalumab + SBRT (8Gy * 1F) Arm C2: Tremelimumab+ Durvalumab + SBRT (5Gy * 5F)	Incidence of adverse events
Genital tumor	NCT03452332	Phase I	Recurrent or Metastatic cervical, vaginal, or vulvar cancers	SABR + Tremelimumab + Durvalumab	Incidence of adverse events
	NCT03795207	Phase II	Prostate cancer with oligometastatic relapse	Arm A: SBRT + Durvalumab Arm B: SBRT	PFS
	NCT03614949	Phase II	Recurrent, persistent, or metastatic cervical cancer	SBRT + Atezolizumab	ORR
	NCT03312114	Phase II	Persistent or recurrent epithelial ovarian, primary peritoneal or fallopian tube cancer	SABR + Avelumab	ORR
Merkel cell cancer	NCT03304639	phase II	Advanced/metastatic Merkel cell carcinoma	Arm A: Pembrolizumab Arm B: Pembrolizumab + SBRT	PFS
	NCT03071406	Phase II	Metastatic Merkel cell carcinoma	Arm A: Nivolumab + Ipilimumab Arm B: Nivolumab + Ipilimumab + SBRT	ORR
Soft tissue cancer	NCT03548428	Phase II	Sarcoma with oligometastases	Arm A: SBRT + Atezolizumab Arm B: SBRT	PFS
	NCT03399552	Phase I/II	Malignant mesothelioma	SBRT + Avelumab	ORR

## Abbreviations

BED: biological effective dose
CTLA-4: cytotoxic T lymphocyte-associated antigen 4
DFS: disease free survival
EDRIC: estimated dose of radiation to immune cells
FFP: freedom from progression
HIF-1α: hypoxia-inducible factor 1α
ICI: immune checkpoint inhibitor
LQ: linear quadratic
LQ-L: linear-quadratic-linear
NSCLC: non-small cell lung cancer
ORR: overall response rate
OS: overall survival
PD1: programmed cell death 1
PD-L1: programmed cell death ligand 1
PDGFR: platelet-derived growth factor
PFS: progression free survival
PLQ: Pade linear quadratic
SABR: stereotactic ablative radiotherapy
SBRT: stereotactic body radiation therapy
SRS: stereotactic radiosurgery
TCP: tumor control probability
TCR: T-cell receptor
USC: universal survival curve
VEGFR: vascular endothelial growth factor receptor
3D-CRT: 3-dimension conformal radiotherapy
